# TRIF Is a Critical Negative Regulator of TLR Agonist Mediated Activation of Dendritic Cells *In Vivo*

**DOI:** 10.1371/journal.pone.0022064

**Published:** 2011-07-08

**Authors:** Sergey S. Seregin, Yasser A. Aldhamen, Daniel M. Appledorn, Charles F. Aylsworth, Sarah Godbehere, Chyong-Jy Joyce Liu, Dionisia Quiroga, Andrea Amalfitano

**Affiliations:** Department of Microbiology and Molecular Genetics, Department of Pediatrics, College of Osteopathic Medicine, Michigan State University, East Lansing, Michigan, United States of America; National Institute of Allergy and Infectious Diseases - Rocky Mountain Laboratories, United States of America

## Abstract

Despite recent advances in developing and licensing adjuvants, there is a great need for more potent formulations to enhance immunogenicity of vaccines. An *Eimeria tenella* derived antigen (rEA) augments immune responses against several pathogens in animal models and recently was confirmed to be safe for human use. In this study, we have analyzed the molecular mechanisms underlying rEA activity in mice, and confirmed that rEA activates multiple immune cell types, including DCs, macrophages, NK, B, and T cells. The rEA adjuvant also elicits the induction of pleiotropic pro-inflammatory cytokines, responses that completely depend upon the presence of the TLR adaptor protein MyD88. Surprisingly, we also found that the TRIF adaptor protein acts as a potent negative regulator of TLR agonist-triggered immune responses. For example, IL12 production and the induction of co-stimulatory molecule expression by DCs and IFNγ production by NK cells *in vivo* were significantly increased in rEA-treated TRIF-KO mice. Importantly, however, TRIF suppressive effects were not restricted to rEA-mediated responses, but were apparent in LPS- or ODN2006-activated DCs as well. Taken together, our findings confirm that rEA is a potent adjuvant, triggering robust activation of the innate immune system, in a manner that is augmented by MyD88 and inhibited by TRIF; thereby unveiling the potential complexities of modulating TLR activity to augment vaccine efficacy.

## Introduction

There is a great need to develop more efficient vaccines to combat or prevent infections by a number of detrimental pathogens that continue to plague mankind [1], [2], [3]. The use of novel adjuvants capable of beneficially stimulating the immune system to maximize efficacy of various vaccination strategies is a rapidly developing field. Most adjuvants augment the induction of innate immune responses by triggering robust activation of dendritic cells (DCs) and macrophages, actions that can result in improved induction of antigen specific adaptive immune responses. Upon migration to the draining lymph nodes, these highly active antigen presenting cells (APCs) are capable of presenting specific antigens to responsive T cells, thereby generating significant pools of antigen-specific T cells [2], [4]. Incorporation of Toll-like receptor (TLR) ligands into vaccine formulations represent a class of adjuvants proposed for usage in next generation vaccines. This is primarily due to TLRs being expressed at high levels on important immune cell types (DCs, macrophages, NK cells) and their ability to potently activate the innate immune system [4], [5], [6].

In light of these facts, the recombinant, *Eimeria tenella* derived antigen (rEA) has been proven to be capable of inducing IL-12p70 production, enhancing Th1 cellular responses, and yielding protection against *Toxoplasma gondii* infection in mice [7]. rEA has also been shown to be an efficient immunomodulator, having both antiviral and anti-cancer properties [8], [9], [10]. Previous studies have also shown that HIV-gag-specific T cell responses are significantly increased when rEA formulations are administered together with the antigen [11], [12]. Moreover, rEA showed no evidence of toxicity in pre-clinical [8] and clinical trials [13]. Specifically, no severe adverse reactions were reported in human clinical trials despite detection of increased IL-12 responses in 30% of the treated cancer patients [13].

The rEA protein has a relatively high amino acid sequence homology (67%) and shares very similar biological activities *in vitro* and *in vivo* with *T. gondii*-derived profilin-like protein, both of which trigger potent IL-12 responses in DCs. The profilin induced responses were completely dependent upon the adaptor protein MyD88 and at least partially mediated via TLR11 [14]. Moreover, it has been shown *in vitro* that human TLRs (TLR2, TLR3, TLR4, TLR5, TLR7, TLR8 and TLR9) do not transduce rEA signaling [9]. Therefore, TLR11 has been suggested as the rEA receptor mediating rEA signaling, but this notion remains to be confirmed. Since no functional human TLR11 homolog has been discovered, these facts leave unidentified the mechanism underlying rEA action in humans, and opens a discussion regarding other pattern recognition receptors (PRRs) that may be involved in rEA signaling [9]. Additionally, it is not known what cell types are primarily responsible for mediating rEA-triggered responses *in vivo*.

MyD88 and TRIF are two adaptor proteins which primarily mediate the signaling derived from activation of many pattern recognition receptors (PRRs), including TLRs [6]. We have investigated if rEA requires either of these two proteins to trigger immune responses *in vivo*, and have found that all rEA-triggered immune responses are dependent on MyD88 functionality (including the rapid activation of DCs, macrophages, NK, NKT, T and B cells, the induction of pro-inflammatory cytokine/chemokine releases, as well as Erk1/2 phosphorylation). Surprisingly, we discovered that functional TRIF protein acts to suppress rEA induction of these same responses; thereby unveiling a novel inhibitory role for TRIF during rEA mediated signaling. We also present evidence that TRIF may similarly suppress TLR activations by other known TLR ligands. Together the findings highlight the complexities underlying adjuvant activation of the innate immune system, as well suggests that simple notions of augmenting or modifying adjuvant activity by use of TLR system agonists or antagonists may be complicated by these complex molecular mechanisms.

## Results

### TRIF acts as a negative regulator of rEA-induced MyD88-dependent activation of dendritic cells and macrophages *in vivo*

**Figure 1:**
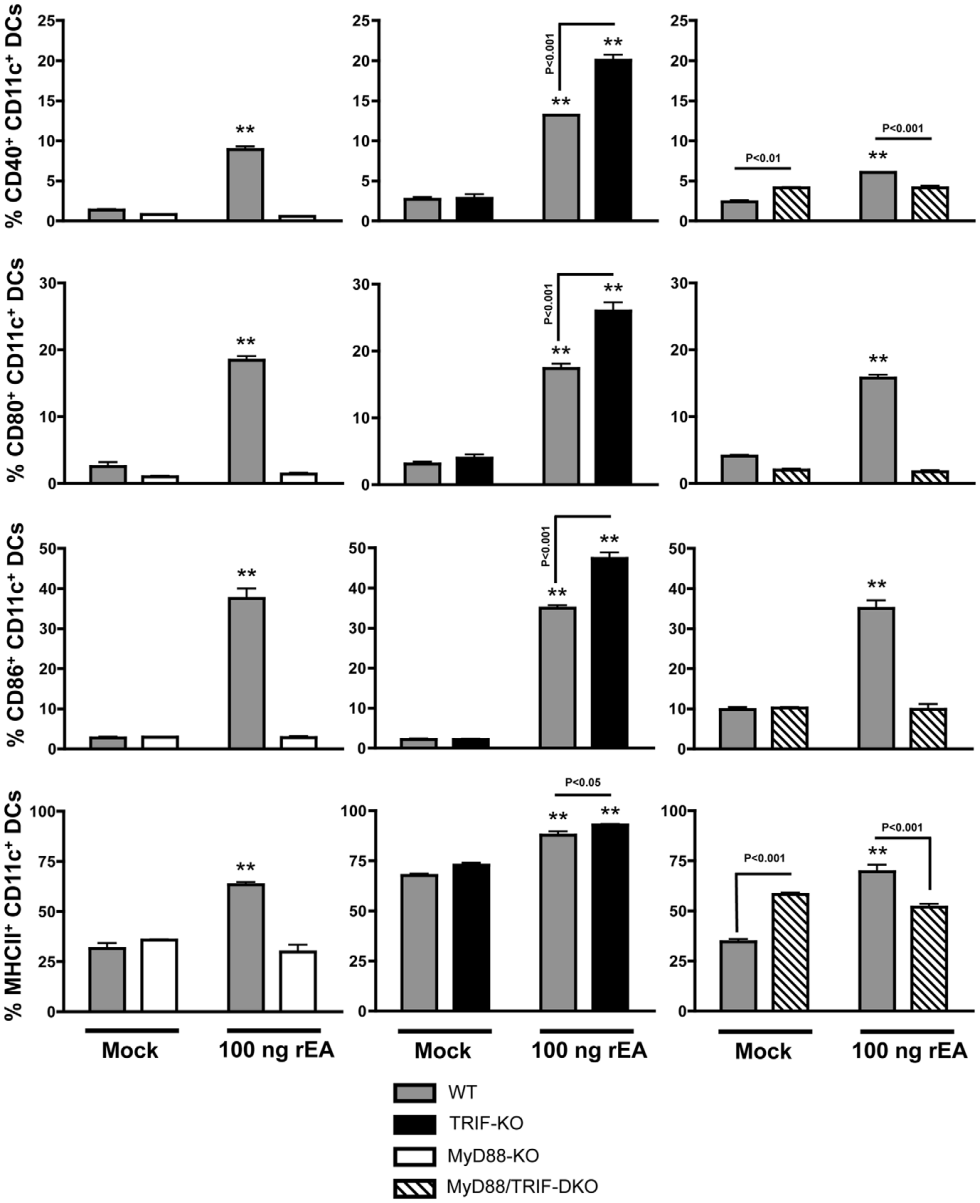
TRIF acts as a negative regulator of rEA-induced MyD88-dependent activation of dendritic cells *in vivo*. C57BL/6 WT (N = 3–4), MyD88-KO (N = 3), TRIF-KO (N = 3–4), and MyD88/TRIF-DKO (N = 4) mice were injected with 100 ng of rEA. Splenocytes were harvested at 6 hpi, processed, stained for expression of surface markers, and FACS sorted as described in Materials and Methods. All genotype mock-injected mice (N = 2–3) were included in analysis. One of two representative experiments is shown. Separate sets of WT mice were utilized for comparison with each knockout genotype. Activation of CD11c^+^, CD19^−^, and CD3^−^ DCs is shown. The bars represent Mean ± SEM. Statistical analysis was completed using a two tailed homoscedastic Student's t-tests. *, ** - Indicate values statistically different from those in mock injected animals (of the same genotype), p<0.05, p<0.001 respectively.

**Figure 2:**
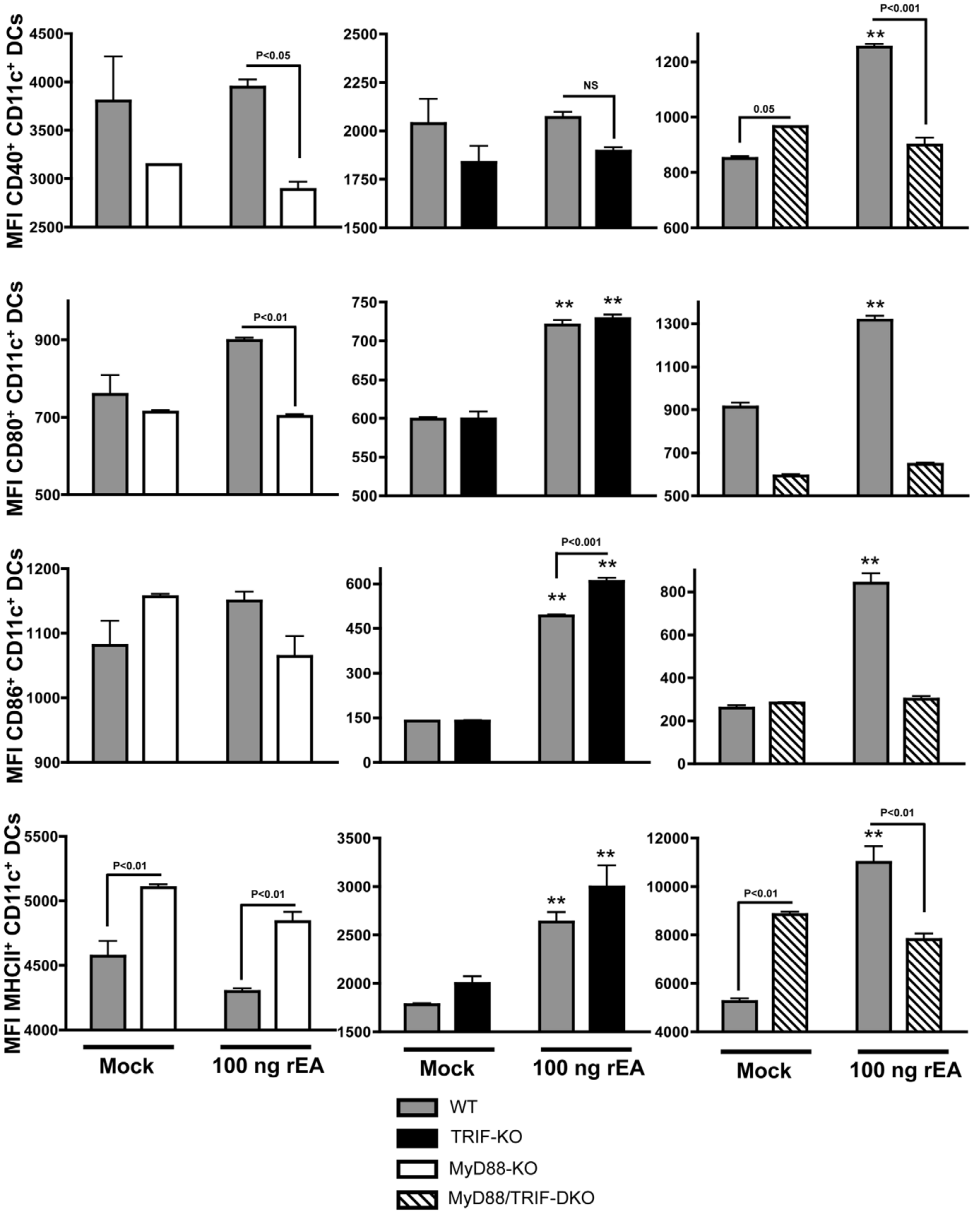
TRIF acts as a negative regulator of rEA-induced MyD88-dependent activation of dendritic cells *in vivo* (MFI). C57BL/6 WT (N = 3–4), MyD88-KO (N = 3), TRIF-KO (N = 3–4), and MyD88/TRIF-DKO (N = 4) mice were injected with 100 ng of rEA. Splenocytes were harvested at 6 hpi, processed, stained for expression of surface markers, and FACS sorted as described in Materials and Methods. All genotype mock-injected mice (N = 2–3) were included in analysis. One of two representative experiments is shown. Separate sets of WT mice were utilized for comparison with each knockout genotype. Mean Fluorescent Intensity (MFI) is shown and is indicative of amount of analyte per cell. The bars represent Mean ± SEM. Statistical analysis was completed using two-tailed homoscedastic Student's t-tests. *, ** - Indicate values statistically different from those in mock-injected animals (of the same genotype), p<0.05, p<0.001 respectively.

The purpose of this study was to identify the impact of rEA stimulation on host immune systems, to define important cell types that respond to or modulate rEA-driven activation, and to identify signaling pathways responsible for activation of the immune system in response to rEA. To investigate this, C57BL/6 mice were each IP injected with 100 ng of purified, rEA protein. Splenocytes were harvested at 6 hpi and flow cytometry was performed as detailed in Materials and Methods. We identified that activation of murine DCs (CD11c^+^, CD19^−^, CD3^−^) in response to rEA was completely dependent on the presence of full MyD88 functionality. Specifically, we found significant increases in the percent of CD40+, CD80+ and CD86+ DCs (as well as induction of the expression of these molecules per cell as measured by Mean Fluorescent Intensity [MFI]) in rEA treated wild type (WT) mice, but no such increases were observed in rEA treated MyD88 knockout (KO) or MyD88/TRIF double knockout (DKO) mice, each as compared to mock-injected animals (Figures 1, 2 and Figures S1, S2). Surprisingly, we detected significant (p<0.001) *increases* in DC activation after identical rEA treatments of TRIF-KO mice (as compared to WT mice), as measured by CD40, CD80 and CD86 surface staining. Furthermore, the amount of CD86 expression per cell was significantly (p<0.001) increased in rEA treated TRIF-KO mice, as compared to rEA treated WT mice (Figures 1, 2 and Figures S1, S2). rEA stimulation also increased the percent of MHC-II presenting DCs in WT and TRIF-KO mice, increases that were not seen in the rEA-treated MyD88-KO or the MyD88/TRIF-DKO mice. The DCs from rEA-treated TRIF-KO mice also had significantly (p<0.05) higher MHC-II surface expression levels as compared to rEA treated WT mice (Figure 1).

rEA-mediated activation of splenic macrophages (CD11b^+^, CD19^−^, CD3^−^) was also completely dependent on MyD88, as confirmed by lack of macrophage activation in response to rEA stimulation in the MyD88-KO or the MyD88/TRIF-DKO mice. In contrast, WT mice injected with rEA experienced a dramatic increase in the levels of CD80, CD86, and MHC-II, on the surface of splenic macrophages (MFI), as well as in the percent of macrophages, expressing the CD40, CD80, and CD86 activation markers. Similar to observations in DCs, macrophages derived from rEA-treated TRIF-KO mice were activated to levels that were significantly *higher* than levels measured in WT mice treated with rEA. Not only were the amounts of CD40-expressing macrophages increased, but also both the percentages of CD80 and CD86 expressing macrophages (p<0.01) and amount of these markers per cell (MFI, p<0.05) were significantly increased in rEA treated TRIF-KO mice as compared to rEA-treated wild type mice, (p<0.05) (Figures S3, S4, S5). These experiments unveiled an important, not previously described, role of TRIF as a suppressor of rEA-induced TLR/MyD88 signaling in DCs and macrophages *in vivo*.

Interestingly, we found that baseline levels of MHC-II expression on splenic DCs were significantly increased in untreated, MyD88-KO and MyD88/TRIF-DKO mice (amount of MHCII per cell, p<0.01, Figure 2) as compared to untreated WT mice or untreated TRIF-KO mice (Figures 1, 2 and Figures S3, S4). Additionally, the MyD88/TRIF-DKO mice also had higher baseline levels of CD40 expression in DCs (Figure 2). This phenomenon was not unexpected, as we had previously noted increased baseline levels of the MHCII β-chain (three-fold higher) in MyD88-KO mice, as confirmed by microarray transcriptome analysis and flow cytometry based analyses ([15] and data not shown). Potentially, these baseline changes may be due to lack of presence of these adaptors during normal mouse development.

### TRIF negatively regulates rEA-mediated activation of NK, NKT, T and B cells *in vivo*

**Figure 3:**
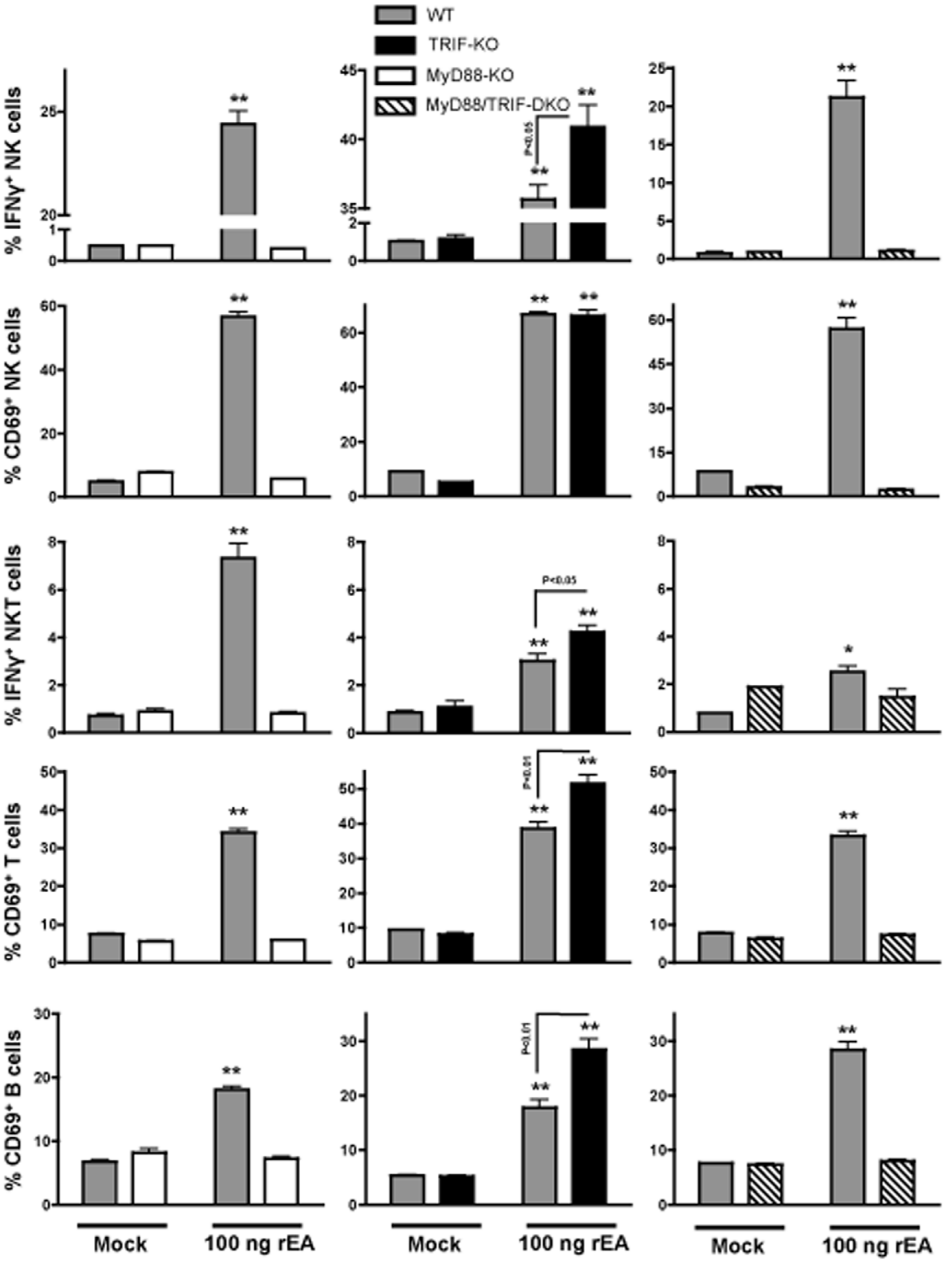
TRIF acts as a negative regulator of rEA-induced MyD88-dependent activation of NK, NKT, T, and B cells *in vivo*. C57BL/6 WT (N = 3–4), MyD88-KO (N = 3), TRIF-KO (N = 3–4), and MyD88/TRIF-DKO (N = 4) mice were injected with 100 ng of rEA. Splenocytes were harvested at 6 hpi, processed, stained for expression of surface markers (intracellular staining was performed for IFNγ), and FACS sorted as described in Materials and Methods. All genotype mock-injected mice (N = 2–3) were included in analysis. Separate sets of WT mice were utilized for comparison with each knockout genotype. Activation of NK, NKT, T, and B cells is shown. The bars represent Mean ± SEM. Statistical analysis was completed using a two tailed homoscedastic Student's t-tests. *, ** - Indicate values statistically different from those in mock injected animals (of the same genotype), p<0.05, p<0.001 respectively.

The unexpected result of TRIF being a negative regulator of rEA-triggered activation of DCs and macrophages, prompted us to evaluate if TRIF reduces rEA-induced activation of other important immune cells, including effector NK cells, as well as NKT, T and B cells. We have previously shown that rEA protein activates splenic and hepatic NK and NKT cells within 6 hours post-injection in WT mice [11]. In this study, rEA injections into MyD88-KO and MyD88/TRIF-DKO mice yielded only baseline activation levels of CD69^+^ NK, NKT, T and B cells. This contrasted with a significant activation of these same cell types in WT mice treated with rEA protein (Figure 3 and Figures S6, S7). Similarly, the amount of IFNγ production from NK and NKT cells was not increased in the MyD88-KO or the MyD88/TRIF-DKO mice in response to rEA, whereas WT mice had dramatically increased numbers of IFNγ secreting NK (from 1% to 20–30%) and NKT (from 1% to 2–5%) cells. In contrast, rEA treatment of TRIF-KO mice revealed significant increases in the number of IFNγ producing NK cells (p<0.05), the amount of CD69 expression per NK cell (MFI, p<0.01), the number of cells (p<0.05) and amount (p<0.05) of IFNγ-production from NKT cells, the number of cells (p<0.01) and amount (p<0.01) of CD69 expression from T cells, and the number of B cells expressing CD69 (p<0.01); all in comparison to rEA-treated WT mice (Figure 3 and Figures S6, S7). Note, rEA stimulation significantly increased the number of CD69-expressing B cells in WT and TRIF-KO mice, but significantly reduced the levels of CD69 expression per cell (Figure S6).

**Figure 4:**
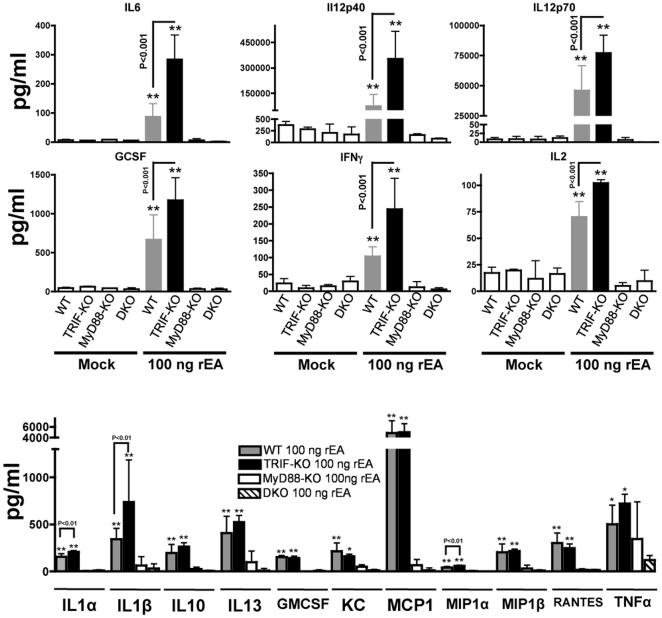
TRIF negatively regulates rEA-mediated MyD88 dependent activation of pro-inflammatory cytokines and chemokines *in vivo*. C57BL/6 WT (N = 9), MyD88-KO (N = 3), TRIF-KO (N = 3–4), and MyD88/TRIF-DKO (N = 4) mice were injected with 100 ng of rEA. Plasma samples were collected at 6 hpi and were analyzed using a multiplexed bead array based quantitative system. All genotype mock-injected mice (N = 2–3) were included in analysis. One of two representative experiments is shown. Statistical analysis was completed using a one-way ANOVA with a Student-Newman-Keuls post-hoc test. The bars represent Mean ± SD. *, ** - Indicate plasma cytokine values that are statistically different from those in mock injected animals, p<0.05, p<0.001 respectively. No significant differences between mock-injected animals of different genotypes were detected. No significant activation of cytokines was observed in MyD88-KO and MyD88/TRIF-DKO animals.

Within 6 hours of administration, rEA protein triggers significant production of pro-inflammatory cytokines and chemokines in mice. Specifically, circulating levels of IL12p70, IL6, TNFα, IFNγ, IL2 were markedly elevated in rEA-treated mice [8], [9]. Purified murine DCs exposed to 0.2 ng/ml of rEA were shown to release significant amounts of IL12p70, IL6, and IL2 [8]. We confirmed and extended the observation that rEA triggers the release of a wide spectrum of pro-inflammatory cytokines and chemokines, including IL6, IL12p40, IL12p70, GCSF, IFNγ, IL2, IL1α, IL1β, IL10, IL13, GMCSF, KC, MCP1, MIP1α, MIP1β, RANTES, and TNFα. This effect was however, completely dependent on MyD88 given that all of these analytes were induced in rEA-treated WT, but not in rEA-treated MyD88-KO or MyD88/TRIF-DKO mice (Figure 4). Again, paralleling our previous results, the release of all of these cytokines and chemokines was significantly increased in rEA-treated TRIF-KO mice, and the majority of these analytes were elevated to levels that were significantly higher than levels measured in rEA-treated WT mice. In particular, IL6 was induced to ∼3 fold (p<0.001) higher levels and IL12p40, GCSF, and IFNγ were induced to over 2 fold (p<0.001) higher levels when comparing rEA-treated TRIF-KO mice to rEA-treated WT mice. Moreover, IL12p70, IL2, IL1α, IL1β, and MIP1α were also produced at significantly higher levels in rEA-treated TRIF-KO mice as compared to rEA treated WT mice (Figure 4).

### Dendritic cells are a major cell type mediating rEA responses

**Figure 5:**
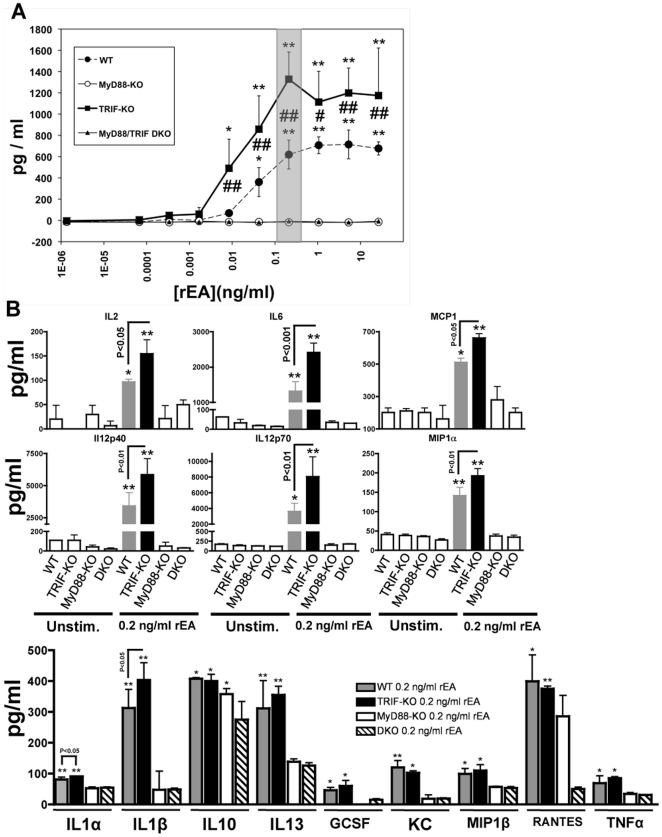
TRIF negatively regulates rEA-mediated MyD88 dependent activation of pro-inflammatory cytokines and chemokines in dendritic cells. (**A**) CD11c^+^ dendritic cells were isolated from C57BL/6 WT (N = 2), MyD88-KO (N = 2), TRIF-KO (N = 2), and MyD88/TRIF-DKO (N = 2) mice, *in vitro* stimulated with rEA, then used to perform a IL12p70 ELISA as described in Materials and Methods. One (of three) representative experiments is shown. The bars represent Mean ± SD. Statistical analysis was completed using a two-way ANOVA with a Bonferroni post-hoc test (genotypes×rEA treatments). *, ** - Indicate values that are statistically different from those in unstimulated DCs (for the same genotype), p<0.05, p<0.001 respectively. #, ## - Indicate values statistically different from those in WT DCs (for the same rEA dose), p<0.05, p<0.01 respectively. No significant differences between mock-injected animals of different genotypes were detected. No significant activation of IL12p70 was observed in MyD88-KO and MyD88/TRIF-DKO DCs. (**B**) DC culture media was collected at 18 hours post-rEA stimulation (0.2 ng/ml) and was analyzed for cytokines/chemokines levels using a multiplexed bead array based quantitative system. Statistical analysis was completed using a one-way ANOVA with a Student-Newman-Keuls post-hoc test. The bars represent Mean ± SD. *, ** - Indicate cytokine values that are statistically different from those in mock injected animals, p<0.05, p<0.001 respectively. No significant differences between mock-injected animals of different genotypes were detected. No significant activation of pro-inflammatory cytokines was observed in MyD88-KO and MyD88/TRIF-DKO animals (only anti-inflammatory IL10 cytokine was induced in MyD88-KO mice).

It is known that many of the rEA induced cytokines and chemokines are released by DCs (Figure 4) [16]. For that reason, we purified CD11c^+^ DCs from WT, MyD88-KO, TRIF-KO, and MyD88/TRIF-DKO mice, and then stimulated the cells *ex vivo* with escalating amounts of rEA protein. Utilizing an IL12p70 specific ELISA, we confirmed that in response to rEA, DC production of this cytokine was completely dependent upon functional MyD88 (Figure 5A). The minimal rEA dose in which WT mouse-derived DCs produced significant amounts of IL12p70 was found to be 100 pg/mL, whereas TRIF-KO mouse-derived DCs only required a 10 pg/mL dose (10 fold less) for significant IL12p70 release. In an overall comparison to WT mouse derived DCs, TRIF-KO mouse derived DCs had a significantly higher production of IL12p70 in response to rEA. The most dramatic difference between these two groups was noted when DCs were stimulated with a 0.2 ng/ml dose of rEA, which caused ∼1300 pg/ml of IL12p70 to be released from DCs derived from TRIF-KO mice as compared to ∼600 pg/ml from DCs derived from WT mice (p<0.01) (Figure 5A.)

We have verified our ELISA-based data for IL12p70 independently, by Bioplex bead array. Again, we confirmed that MyD88-KO and the MyD88/TRIF-DKO mouse derived DCs each respectively failed to produce significant levels of pro-inflammatory cytokines and chemokines in response to rEA stimulation. Interestingly, we also confirmed that these rEA mediated DC responses were also partially suppressed by TRIF, as, IL2, IL6, IL12p40, IL12p70, IL1α, IL1β, and MIP1α were released to significantly (p<0.05) higher levels in rEA-treated DCs derived from TRIF-KO mice, as compared to those derived from WT mice (Figure 5B).

### TRIF negatively regulates cytokine production by DCs, triggered by several common TLR agonists

**Figure 6:**
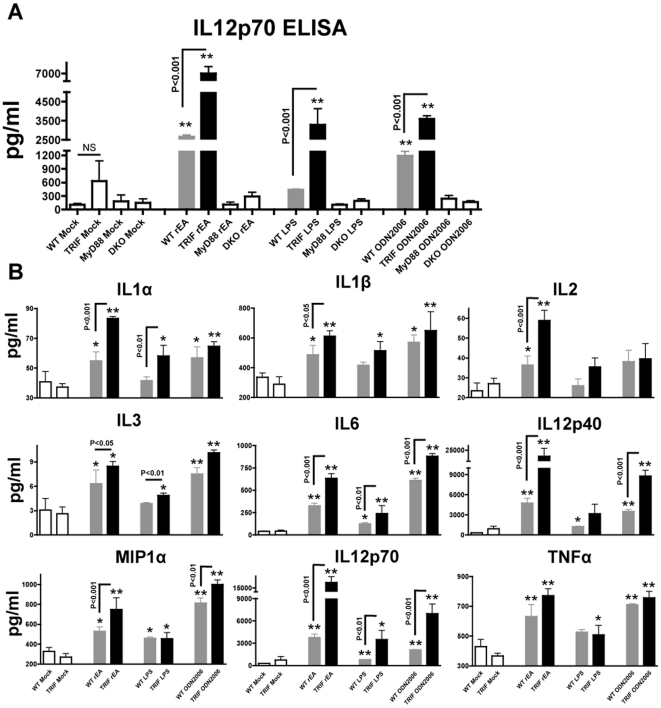
TRIF negatively regulates cytokine production by DCs, triggered by several common TLR agonists. (**A**) CD11c^+^ dendritic cells were isolated from C57BL/6 WT (N = 3), MyD88-KO (N = 3), TRIF-KO (N = 3), and MyD88/TRIF-DKO (N = 3) mice, *in vitro* stimulated with various TLR agonists, and used to perform a IL12p70 ELISA as described in Materials and Methods. The bars represent Mean ± SEM. Statistical analysis was completed using a one-way ANOVA with Student-Newman-Keuls post-hoc test. ** - Indicate values that are statistically different from those in unstimulated DCs (for the same genotype), p<0.001. No significant differences between mock-injected animals of different genotypes were detected. No significant activation of IL12p70 was observed in MyD88-KO and MyD88/TRIF-DKO DCs. (**B**) DC culture media was collected at 15 hours post stimulation with various TLR agonists (rEA, LPS, ODN2006) and was analyzed for cytokines/chemokines levels using a multiplexed bead array based quantitative system. Statistical analysis was completed using a one-way ANOVA with a Student-Newman-Keuls post-hoc test. The bars represent Mean ± SD. *, ** - Indicate cytokine values that are statistically different from those in mock injected animals, p<0.05, p<0.001 respectively.

To more fully investigate if TRIF suppressive effects were rEA-specific or a more global phenomenon, we have specifically stimulated CD11c^+^ DCs, isolated from WT, TRIF-KO, MyD88-KO or MyD88/TRIF-DKO mice with various common TLR4, TLR7/8, an TLR9 agonists. Specifically, LPS, R848 and ODN2006 were utilized in these experiments. All of these TLR agonists were able to induce pro-inflammatory cytokine production after administration to DCs, as compared to unstimulated DCs, all derived from WT mice. Importantly, however, DCs derived from TRIF-KO mice had dramatically higher levels of secretion of pleiotropic pro-inflammatory cytokines, when stimulated with these same TLR agonists (Figure 6AB). Specifically, ODN2006 stimulation resulted in significantly higher IL6, IL12p40, IL12p70 and MIP1α levels; LPS stimulation significantly increased IL1α, IL3, IL6, IL12p70 levels; R848 treatment resulted in significantly higher production of IL1α, IL1β, IL6, and MIP1α; in DCs, derived from TRIF-KO mice as compared to DCs derived from WT mice, as tested both by ELISA (Figure 6A) and Bioplex analysis (Figure 6B and data not shown). DCs derived from MyD88-KO or MyD88/TRIF-DKO mice did not show any significant cytokine activations when stimulated with any of these TLR agonists (data not shown). Interestingly, lack of the TRIF adaptor protein resulted in up to 2 fold increases in cytokine production from DCs, when stimulated with LPS or ODN2006 (i.e. IL12p70), indicating that the suppressive role of TRIF in this cell type might be immunologically significant.

### rEA-triggered Erk1/2 phosphorylation is MyD88 dependent

**Figure 7:**
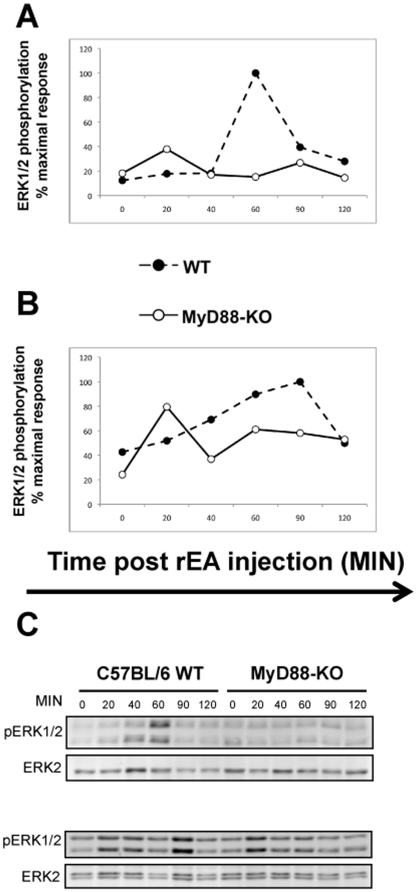
rEA-triggered Erk1/2 phosphorylation is MyD88 dependent. C57BL/6 WT or MyD88-KO mice were injected with 100 ng of rEA. Spleen (**A**) and liver (**B**) tissues were collected at the indicated time points and processed as described in Materials and Methods. p-Erk1/2 and Erk2 levels were determined by Western blot analysis using LI-COR Odyssey. To control for loading, quantification was performed after normalizing the pErk1/2 to Erk2 levels. Three independent experiments representative of this data are shown. (**C**) Representative blots: spleen (top), liver (bottom).

Extensive past research has demonstrated that the major signaling pathways activated downstream of TLR adaptor proteins (e.g. MyD88, TRIF) are the NFκB and MAPK pathways [17]. Along with many other biological roles, these pathways promote production of cytokines and chemokines as well as proliferation, maturation, and development of various immune cells. To identify which of the several signaling pathways may be activated in response to rEA *in vivo*, we IP injected WT or MyD88-KO mice with 100 ng of rEA, then collected spleen and liver tissues at various time points (0–120 minutes) post-injection. We found that rEA-triggered Erk1/2 phosphorylation was MyD88 dependent, as confirmed by increased Erk1/2 phosphorylation in rEA-treated WT mice, but not in the MyD88-KO mice (Figure 7).

## Discussion

The ideal adjuvant (from Latin “*adjuvare*”, meaning “to enhance”) is an agent that is capable of dramatically enhancing both cellular and humoral adaptive immune responses to co-administered antigens, thereby providing more efficient and long-term protection against specific pathogens. Aluminum salts, discovered to be potent adjuvants in 1920s, remained the only FDA approved adjuvant for many decades and still represent one of the few in clinical use. A barrier in adjuvant research has been that the mechanism of action of many adjuvants remained poorly understood. Earlier studies showed that alum and squalene-based emulsion MF59 promote recruitment and increase antigen uptake by APCs, induce cytokine and chemokine secretions, and the expression of adhesion molecules involved in migration of leukocytes [18], [19]. More recently it has also been confirmed that alum and squalene based adjuvants may use the NOD-like receptor protein 3 (NLRP3) inflammasome pathway to activate the innate immune system [20], [21].

The *Eimeria tenella* derived protein, was isolated from bovine small intestinal extracts and was shown to have remarkable anti-cancer activity and be a potent stimulator of innate immune responses in various mouse models *in vitro* and *in vivo* [8], [22], [23]. The rEA protein, we believe, might possess all the properties of an ideal immunologic adjuvant, as it can be fairly inexpensive to produce, is extremely stable and can be stored for long periods of time (over 24 month, data not shown) without losing activity. The rEA has been shown to be safe and very well-tolerated in human clinical trials [13]. In mice, rEA augments activation of the innate immune system, presumably by activating PRRs (TLRs and/or possibly others) and thereby increasing adaptive immune responses to co-administered antigens [11], [12]. It has been suggested that TLR11 is involved in rEA signaling [9], a notion that is primarily based on high sequence homology (67%) between rEA and *T. gondii* profilin-like protein, the latter being the only confirmed ligand for TLR11 [14].

In this study we confirmed that administration into mice, or direct exposure of immune cells to rEA protein results in (1) activation of important immune cell types (DCs, macrophages, NK, NKT, B and T cells), including (2) pro-inflammatory cytokines/chemokines release, both globally and specifically by DCs, and highly robust IFNγ production by NK cells, and (3) Erk1/2 phosphorylation. Therefore, we showed that rEA, similarly to other TLR-agonist-based adjuvants, activated an innate immune profile that resulted in robust activation of innate immune cells and induced multiple cytokines/chemokine pathways. Our results also confirmed that these responses are completely dependent upon MyD88, as genetic knockout of this adaptor protein results in complete ablation of these responses both *in vitro* and *in vivo*.

When we similarly investigated the role of TRIF, the other major TLR adaptor protein, we encountered unexpected results. Specifically, TRIF-KO mice showed dramatic *increases*, in immune cell activation and other rEA triggered responses, when compared to rEA treated WT mice. Specifically, we found that IL6, IL12p40, IL2, IL1α, IL1β, and MIP1α production was induced by rEA treatment in TRIF-KO mice *in vivo* and in DCs derived from these mice *in vitro* to significantly higher levels as compared to similar assays performed in rEA-treated WT mice. Note, that rEA mediated induction of IL12p70, a cytokine abundantly produced by DCs that activates NK cells, was also found to be negatively regulated by TRIF after exposure to rEA [16], [24].

Therefore, in response to rEA-mediated stimulation, TRIF acts as a suppressor of the rEA-induced (MyD88-dependent) activation of DCs, macrophages, NK, NKT, T, and B cells *in vivo*. Despite the lack of TRIF activity in MyD88/TRIF-DKO mice, rEA responses were still ablated which indicates that in the absence of MyD88, TRIF cannot carry out its suppressive activity. Since the lack of TRIF protein does not rescue the phenotype in MyD88/TRIF-DKO mice, this confirms an essential role of MyD88 in mediating these responses and suggests that TRIF protein acts as a suppressor *downstream* of MyD88 and/or MyD88 and TRIF adaptor molecules orchestrating the induction of pro-inflammatory immune responses following rEA injection.

Numerous studies have described important roles for TRIF as a TLR system adaptor protein that acts to enhance TLR-based signaling (predominantly when TLR3 and TLR4 ligands are interrogated, thereby promoting antimicrobial responses [6], [17], [25], [26]. Specifically, TRIF-KO mice showed dramatically impaired lung clearance of *Pseudomonas aeruginosa* infections; a response that is correlated with blunted cytokine induction (e.g. RANTES, IL1β, MIP2) and reduced NFκB activation present in both alveolar and peritoneal macrophages from these mice [27]. Lack of TRIF protein also results in reduced induction of antigen specific humoral and cellular immune responses in other models [28]. Specifically, T cells derived from TRIF-KO mice had dramatically reduced IFNγ production and CXCR3 expression upon antigen/LPS treatment as compared to WT mice [29]. In DCs, TRIF functionality was confirmed to be important for upregulation of CD40 and CD86 co-stimulatory molecules [30]. Moreover, TRIF protein along with IPS1 (RIGI/Mda5 pathway protein) are key adaptors in mediating PolyIC-triggered adjuvant effects [28].

Conversely, there is very little data available that describes suppressive roles of TRIF protein on TLR/PRR signaling. Specifically, we have previously demonstrated that a lack of functional TRIF protein results in increased transgene (e.g. β-Gal) specific IgG titers in mice injected with Ad5-LacZ, suggesting that TRIF may act as a negative regulator of Ad-mediated antibody responses in mice [31]. Other researchers have demonstrated that TRIF has an inhibitory role in TLR5-mediated responses through induction of TLR5 degradation [32]. This phenomenon may not be limited to TLR5, as it has been suggested that TRIF can also induce degradation of other TLRs, including TLRs 3, 6, 7, 8, 9, and 10 [32]. To determine if the suppressive role of TRIF in DCs is more global than previously considered, we have stimulated isolated DCs with TLR4 (LPS), TLR7/8 (R848) or TLR9 (ODN2006) agonists and found that the presence of the TRIF adaptor protein significantly *suppresses* release of pleiotropic pro-inflammatory cytokines by DCs in response to these agonists. These studies suggest that suppressive activities of TRIF, in regard to pathogen-induced innate immune responses, may be more prevalent than currently appreciated.

Activation of pro-inflammatory cytokines/chemokines is a critical step during DC activation/maturation. It has been suggested that TRIF may be required for inducing immunological tolerance by augmenting IL10 production [33], [34]. We found, however, that in rEA (or other TLR agonist) treated TRIF-KO mice, amounts of IL10 (both in plasma and in isolated DCs) were indistinguishable to identically (rEA) treated WT mice.

To successfully bridge DC activation to induction of substantial adaptive immune responses (e.g. T cell activation), it is essential to have induction of co-stimulatory molecules such as CD40, CD80, and CD86 on the surface of DCs. In this study, we have shown that administration of rEA results in robust maturation of DCs, as evidenced by not only increased expression of co-stimulatory molecules on these DCs, but also that rEA increased cytokines and chemokine production. While these responses were completely abrogated in rEA-treated MyD88-KO mice, most of them were dramatically enhanced in TRIF-KO mice when compared to rEA treated WT mice.

**Figure 8:**
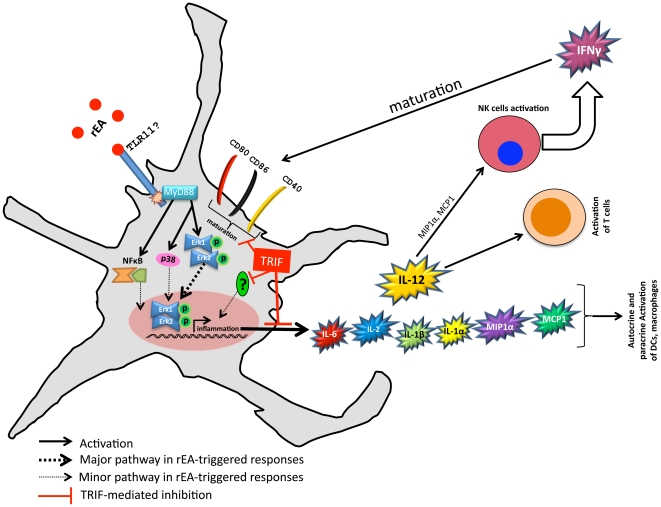
TRIF acts as a negative regulator of rEA-induced signaling and downstream responses in DCs: model of action. rEA is a protein derived from *Eimeria tenella* [8], [11] and is highly homologous to *Toxoplasma gondii* profilin-like protein [14]. *T. gondii* has been shown to signal, at least in part, through TLR11; therefore, it is likely that TLR11 is one of the main pattern recognition receptors (PRRs) utilized by rEA. We have shown that rEA-triggered responses *in vivo* are completely dependent on MyD88. MAP kinases consequently become activated downstream of MyD88. Importantly, functional TRIF protein inhibited rEA-mediated signaling. DCs are a major cell type in mediating rEA responses and, under TRIF's inhibitory effects, have mitigated induction of surface expression of maturation markers and stunted release of pro-inflammatory cytokines/chemokines. Release of these molecules is critical for rapid amplification of immune responses and is mediated by autocrine and paracrine signaling [16]. We have confirmed that TRIF protein reduces release of pro-inflammatory cytokines/chemokines in response to rEA, resulting in reduced activation of NK, NKT, T, and B cells as well as reduced IFNγ production by NK cells.

From this information, we are proposing a model of rEA signaling in DCs (see Figure 8). The rEA protein likely interacts with a PRR, (likely TLR11 in mice, unknown in humans [9]), that senses rEA. The MyD88 adaptor protein gets recruited which allows MAP kinases downstream of MyD88 to become activated (pErk1/2, p38). pErk1/2 is capable of activating various transcription factors (e.g. AP1) which further activates pro-inflammatory genes, including those of pro-inflammatory cytokines/chemokines [17], [26], [29], [35]. In contrast, functional TRIF protein acts as a negative regulator of the rEA-induced signaling. As a result of TRIF's inhibitory effects, DCs have a reduction in surface expression of maturation markers, as well mitigated release of pro-inflammatory cytokines/chemokines [16].

In conclusion, our murine models have shown that rEA activates multiple immune cell types, stimulates pro-inflammatory cytokine/chemokine release, and triggers the MAPK pathway in a MyD88 dependent manner. We have also confirmed that DCs are the main subset of innate immune cells that mediate rEA-triggered responses, thus justifying future studies on isolated DCs and the potential use of rEA adjuvant in a DC-vaccine setting [36]. Importantly, our studies of rEA unveiled a suppressive activity to the TRIF adaptor protein. This clearly justifies future testing of specific TRIF inhibitors or knockdown models prior to rEA/antigen administration as a means to further enhance induction of antigen specific adaptive immune responses. Whether TRIF acts to negatively regulate other adjuvants or TLR-mediated activations is a question that will require future investigations. The latter, however, highlights the complexities of TLR adaptor functions, and should temper efforts that target these proteins with agonists or antagonists, as the end result of such interventions may run counter to hoped for outcomes, and could be detrimental in some situations [37].

## Materials and Methods

### Animal Procedures

All animal procedures were reviewed and approved by the Michigan State University ORCBS and IACUC. Care for mice was provided in accordance with PHS and AAALAC standards (ID number: A3955-01). Adult male C57BL/6 mice were purchased from The Jackson Laboratory (Bar Harbor, ME). MyD88-KO and TRIF-KO mice were kindly provided by Dr. Shizuo Akira. MyD88/TRIF-DKO mice were bred at Michigan State University. Intraperitoneal (IP) injection of animals (2–4 months in age) consisted of 100 µl phosphate-buffered saline solution (PBS, pH 7.4) containing 100 ng rEA protein from Eimeria *tenella* as previously described [8], [11]. rEA protein purification was performed as previously described [8] with minor modifications described [11]. Plasma and tissue samples were obtained and processed at the indicated times post-injection as previously described [11]. Importantly, IP route of rEA administration was confirmed to be more efficient that other widely used routes for adjuvant injection, such as intranasal or subcutaneous [7], [8]. To study rEA-triggered activation of immune cells by flow cytometry and bead array methods, plasma and spleen tissues were harvested at 6 hours post injection (hpi), whereas for studies measuring activation of signaling pathways the spleen and liver tissues were harvested at 0, 20, 40, 60, 90, and 120 minutes post-rEA injection.

### Cytokine and chemokine analysis

A mouse 23-plex multiplex based assay was used to determine the indicated cytokine/chemokine concentrations in the plasma collected *in vivo* or the media collected from cultured DCs per the manufacturer's instructions (Bio-Rad, Hercules, CA) via Luminex 100 technology (Luminex, Austin, TX) as previously described [38].

### Isolation of Splenocytes

Splenocytes from individual mice were harvested and processed by physically disrupting and facilitating passage of splenic tissue through a 40 µm sieve, followed by induction of RBC lysis by using 2 ml of ACK lysis buffer (Invitrogen, Carlsbad, CA) per homogenized spleen. Splenocytes were subsequently washed two times with RPMI medium 1640 (Invitrogen, Carlsbad, CA) supplemented with 10% FBS, 2 mM L-glutamine, and 1% PSF (penicillin, streptomycin, fungizone), then resuspended and counted.

### Cell staining and flow cytometry

Splenocyte preparations were evaluated for the presence of DC, macrophages, NK, NKT, T, and B cell activation/maturation markers as previously described [11], [39]. 1×10^6^ cells were stained with combinations of the following antibodies: APC-CD3, PerCpCy5.5-CD19, PE-Cy7-NK1.1, and PE-CD69 (all 4 µg/ml) or PECy7-Cd11c, APCCy7-CD11b, APC-CD80, Pacific Blue-CD86, Alexa Fluor700-MHCII, FITC-CD40, and PerCpCy5.5-CD3/CD19/NK1.1 (dump channel) (all 4 µg/ml) (BD Biosciences, San Diego, CA). Cells were incubated on ice with the appropriate antibodies in 2.4G2 hybridoma cell supernatant for 30 minutes, then washed and sorted using a BD LSR II instrument. For intracellular staining, 2×10^6^ splenocytes were incubated with Brefeldin A (1 µg/ml) in DMEM with 10%FBS/1×PSF for 4 hours at 37°C. Following incubation, splenocytes were washed two times with FACS buffer, incubated for 15 minutes with purified rat anti-mouse CD16/CD32 Fcγ block (BD Biosciences, San Diego, CA), surface stained with CD3-APC and NK1.1-PECy7 (8 µg/ml) for 30 minutes at 4° C, washed with FACS buffer, fixed with 2% formaldehyde (Polysciences, Warrington, PA) for 20 minutes on ice, permeabilized with 0.5% Saponin (Sigma-Aldrich, St. Louis, MO) for 20 minutes at room temperature, and incubated on ice with IFNγ-FITC (8 µg/ml) for 2 hours. Samples were analyzed on a BD LSR II instrument using FlowJo software (Tree Star, San Carlos, CA, USA).

### Western blotting

Spleen and liver tissues were homogenized in lysis buffer (20 mM Tris-HCl, pH 7.4, 1 mM EDTA, 150 mM NaCl) containing 1% Triton X-100 with protease inhibitors. Homogenized tissues were then centrifuged at maximum speed (13,000×G) for 10 min at 4°C, after which the protein concentration of the supernatant determined using BCA method. Western blotting for pErk1/2 and Erk2 was performed as previously described [40], [41], [42]. Equivalent concentrations of protein samples were run on polyacrylamide gels and transferred onto nitrocellulose membranes. Blots were then probed with fluorescent antibodies as previously described, which included antibodies against pErk1/2 (Cell Signaling, Inc., Boston, MA) and Erk2 (Santa Cruz Biotechnologies, Santa Cruz, CA). Blots were scanned and bands were quantified using Licor's Odyssey scanner [41]. For data analysis, the fluorescence of pErk1/2 bands was normalized to Erk2 bands prior to quantification.

### Mouse Dendritic Cells isolation and mIL12p70 ELISA

An *in vitro* bioassay originally developed to monitor rEA activity during its purification and production, as well as provide some insight into the mechanism of action of rEA, was previously described [8]. The assay is based upon monitoring the release of mIL12(p70) from mouse DCs as an indicator of rEA-induced DC activation. Mouse DCs (CD11c^+^ splenocytes) were isolated from C57BL/6 WT, MyD88-KO, TRIF-KO, or MyD88/TRIF-DKO mice using the magnetic assisted cell separation (MACS) system (Miltenyi Biotech, Auburn, CA) utilizing the established protocols from the manufacturer and previously described [8]. DC sorting via flow cytometry resulted in >95% pure CD11c positive cell population (Figure S8). The viability of recovered DCs was ∼90%, as measured by trypan blue viability staining.

Isolated CD11c^+^ cells were seeded at a density of 0.3×10^5^ cells per well into 96-well plates in 200 µl/well complete medium. The complete medium consisted of DMEM/F12 supplemented with 10% FCS, gentamicin (10 µg/ml), recombinant mouse GMCSF (1 ng/ml), recombinant mouse IL4 (1 ng/ml), recombinant mouse IFNγ (3 ng/ml), and an agonistic anti mouse CD40 antibody (0.5 mg/ml) (R&D Systems, Minneapolis, MN). This synergistic combination of cytokines has no significant effect on mouse IL12 release, but dramatically enhances the inducing effects of rEA on mouse IL12 release [8]. Mouse DCs were stimulated with rEA (10^−6^–10^1^ ng/ml) overnight (∼18 hours) at 37° C in 5% CO_2_ and 95% ambient air. To study the specificity of TRIF inhibitory effects in DCs, various toll-like receptor agonists were added to mouse DCs, using the following concentrations: rEA (Barros Research Institute, Holt, MI, 100 ng/ml); E. *coli* 0111.B4 LPS (20 µg/ml); R848 (0.5 µg/ml) and ODN2006 (2.5 µM). LPS, R848 and ODN2006 (TLR4, TLR7/8 and TLR9 agonists respectively) were purchased from InvivoGen, San Diego, CA, reconstituted with endotoxin-free water and diluted in culture media (DMEM/F12+10% FCS). Following incubation, culture medium was analyzed for mouse IL12p70 levels using an ELISA kit and following its enclosed instructions (R&D Systems, Minneapolis, MN) or for 23 mouse cytokines/chemokines using multiplex system (Bio-Rad, Hercules, CA).

### Statistical analysis

Statistically significant differences in immune cells activation assays were determined using two-tailed homoscedastic Student's t-tests to compare rEA mediated responses in WT mice against each individual knockout genotype in independent experiments (p value<0.05). Statistically significant differences in plasma or media cytokine levels were determined using a one-way ANOVA with a Student-Newman-Keuls post-hoc test (p value<0.05). Furthermore, a two-way ANOVA with a Bonferroni post-hoc test was used to analyze the levels of IL12p70 in media from cultured DCs (genotype×rEA treatments) that were derived from WT, MyD88-KO, TRIF-KO or MyD88/TRIF-DKO mice and treated with several escalating concentrations of rEA. Graphs in this paper are presented as mean of the average ± SEM, unless otherwise specified. GraphPad Prism software was utilized for statistical analysis.

## Supporting Information

Figure S1 **TRIF acts as a negative regulator of rEA-induced MyD88-dependent activation of dendritic cells** ***in vivo*** **.** Representative histograms are illustrated on this figure.
(TIF)

Figure S2 **Sorting strategy for CD11c^+^ cells.** Representative figure of the gating strategy for sorting CD11c+ DCs. (**A**) Splenocytes were stained with CD11c-PECy7 and CD11b-APC-Cy7 antibodies and data were generated using LSR-II cytometer. (**B**) Splenocytes were stained with CD11c-PECy7 and PerCPCy5.5 conjugated antibodies for CD3, NK1.1, and CD19 to exclude T-, NK-, and B-cells from the analysis.
(TIF)

Figure S3 **TRIF acts as a negative regulator of rEA-induced MyD88-dependent activation of macrophages** ***in vivo*** **.** C57BL/6 WT (N = 3–4), MyD88-KO (N = 3), TRIF-KO (N = 3–4), and MyD88/TRIF-DKO (N = 4) mice were injected with 100 ng of rEA. Splenocytes were harvested at 6 hpi, processed, stained for expression of surface markers, and FACS sorted as described in Materials and Methods. All genotype mock-injected mice (N = 2–3) were included in analysis. One of two representative experiments is shown. Separate sets of WT mice were utilized for comparison with each knockout genotype. Activation of CD11b^+^, CD19^−^, and CD3^−^ macrophages is shown. The bars represent Mean ± SEM. Statistical analysis was completed using a two tailed homoscedastic Student's t-tests. *, ** - Indicate values significantly higher (#, ## - lower) from those in mock injected animals (of the same genotype), p<0.05, p<0.001 respectively.
(TIF)

Figure S4 **TRIF acts as a negative regulator of rEA-induced MyD88-dependent activation of macrophages cells** ***in vivo*** **.** C57BL/6 WT (N = 3), MyD88-KO (N = 3), TRIF-KO (N = 3), and MyD88/TRIF-DKO (N = 4) mice were injected with 100 ng of rEA. Splenocytes were harvested at 6 hpi, processed, stained for expression of surface markers, and FACS sorted as described in Materials and Methods. All genotype mock-injected mice (N = 2–3) were included in analysis. Separate sets of WT mice were utilized for comparison with each knockout genotype. Mean Fluorescent Intensity (MFI) is shown and is indicative of amount of analyte per cell. The bars represent Mean ± SEM. Statistical analysis was completed using two-tailed homoscedastic Student's t-tests. *, ** - Indicate values significantly higher (#, ## - lower) from those in mock-injected animals (of the same genotype), p<0.05, p<0.001 respectively.
(TIF)

Figure S5 **TRIF acts as a negative regulator of rEA-induced MyD88-dependent activation of macrophages** ***in vivo*** **.** Representative histograms are illustrated on this figure.
(TIF)

Figure S6 **TRIF acts as a negative regulator of rEA-induced MyD88-dependent activation of NK, NKT, T, and B cells** ***in vivo*** **.** C57BL/6 WT (N = 3), MyD88-KO (N = 3), TRIF-KO (N = 3), and MyD88/TRIF-DKO (N = 4) mice were injected with 100 ng of rEA. Splenocytes were harvested at 6 hpi, processed, stained for expression of surface markers (intracellular staining was performed for IFNγ), and FACS sorted as described in Materials and Methods. All genotype mock-injected mice (N = 2–3) were included in analysis. Separate sets of WT mice were utilized for comparison with each knockout genotype. Mean Fluorescent Intensity (MFI) is shown and is indicative of amount of analyte per cell. The bars represent Mean ± SEM. Statistical analysis was completed using two-tailed homoscedastic Student's t-tests. *, ** - Indicate values significantly higher (#, ## - lower) from those in mock-injected animals (of the same genotype), p<0.05, p<0.001 respectively.
(TIF)

Figure S7 **TRIF acts as a negative regulator of rEA-induced MyD88-dependent activation of NK, NKT, T, and B cells** ***in vivo*** **.** Representative histograms and plots are illustrated on this figure.
(TIF)

Figure S8 **Validation of CD11c+ DCs isolation.** DC sorting resulted in >95% pure CD11c positive cell population. The viability of recovered DCs was ∼90% as measured by trypan blue viability staining.
(TIF)
