# Supplementary figures and images for: TRIF Is a Critical Negative Regulator of TLR Agonist Mediated Activation of Dendritic Cells In Vivo
Source: PLoS One. 2011 Jul 8;6(7):e22064. doi: 10.1371/journal.pone.0022064 (PMC3132756; doi:10.1371/journal.pone.0022064)

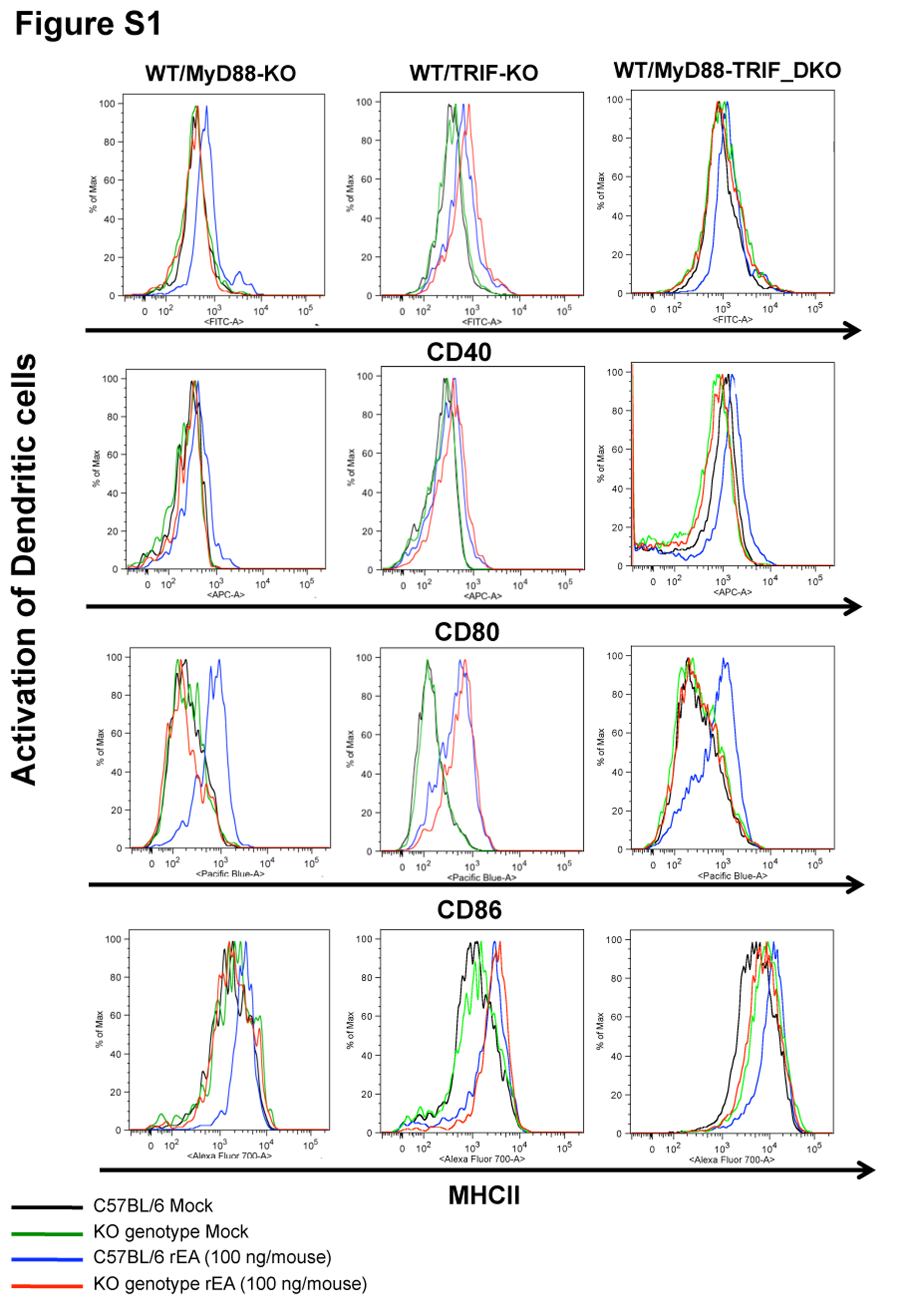

Supplement: Figure S1 — TRIF acts as a negative regulator of rEA-induced MyD88-dependent activation of dendritic cells in vivo . Representative histograms are illustrated on this figure. (TIF) [file pone.0022064.s001.tif]

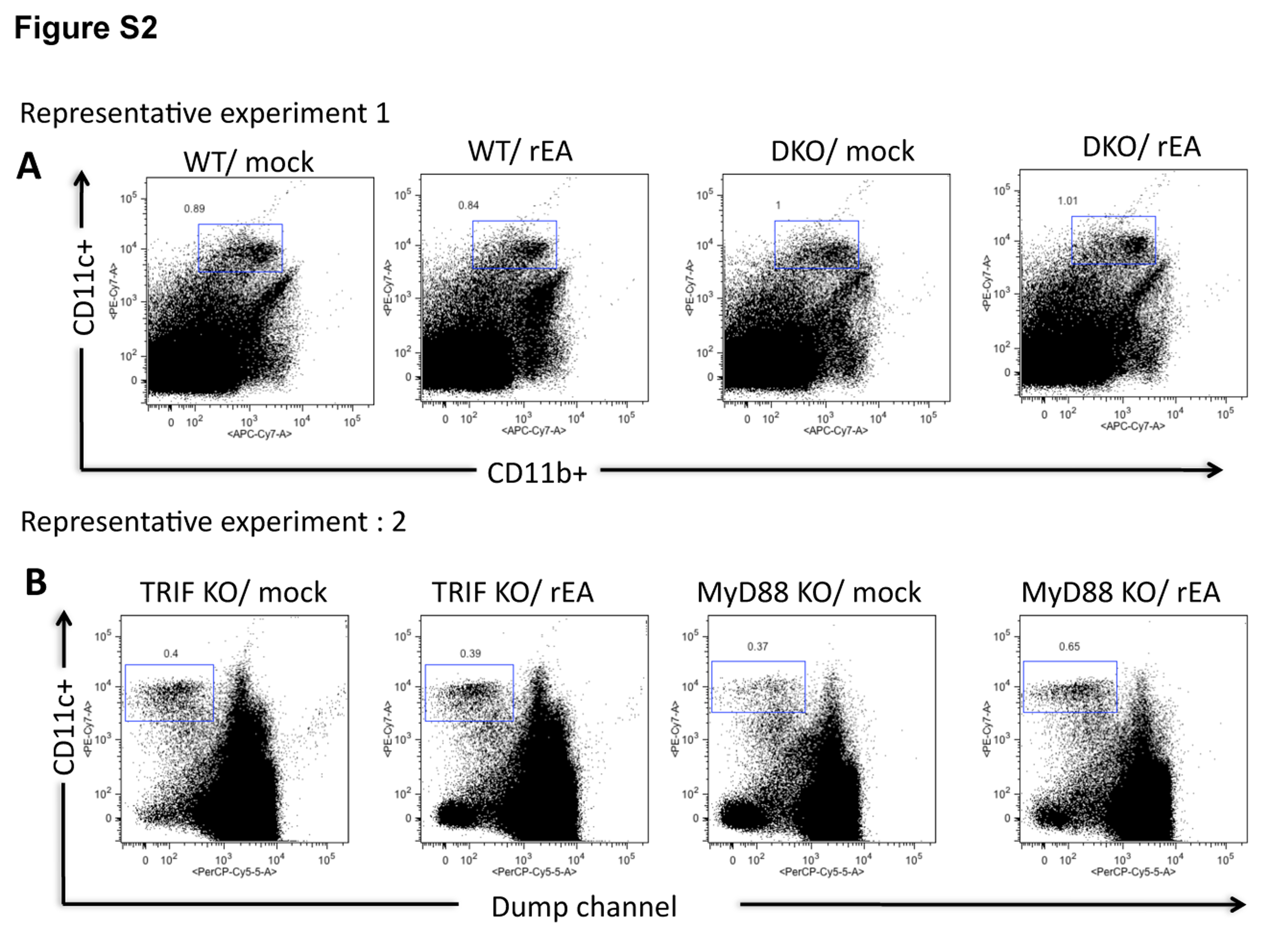

Supplement: Figure S2 — Sorting strategy for CD11c+ cells. Representative figure of the gating strategy for sorting CD11c+ DCs. (A) Splenocytes were stained with CD11c-PECy7 and CD11b-APC-Cy7 antibodies and data were generated using LSR-II cytometer. (B) Splenocytes were stained with CD11c-PECy7 and PerCPCy5.5 conjugated antibodies for CD3, NK1.1, and CD19 to exclude T-, NK-, and B-cells from the analysis. (TIF) [file pone.0022064.s002.tif]

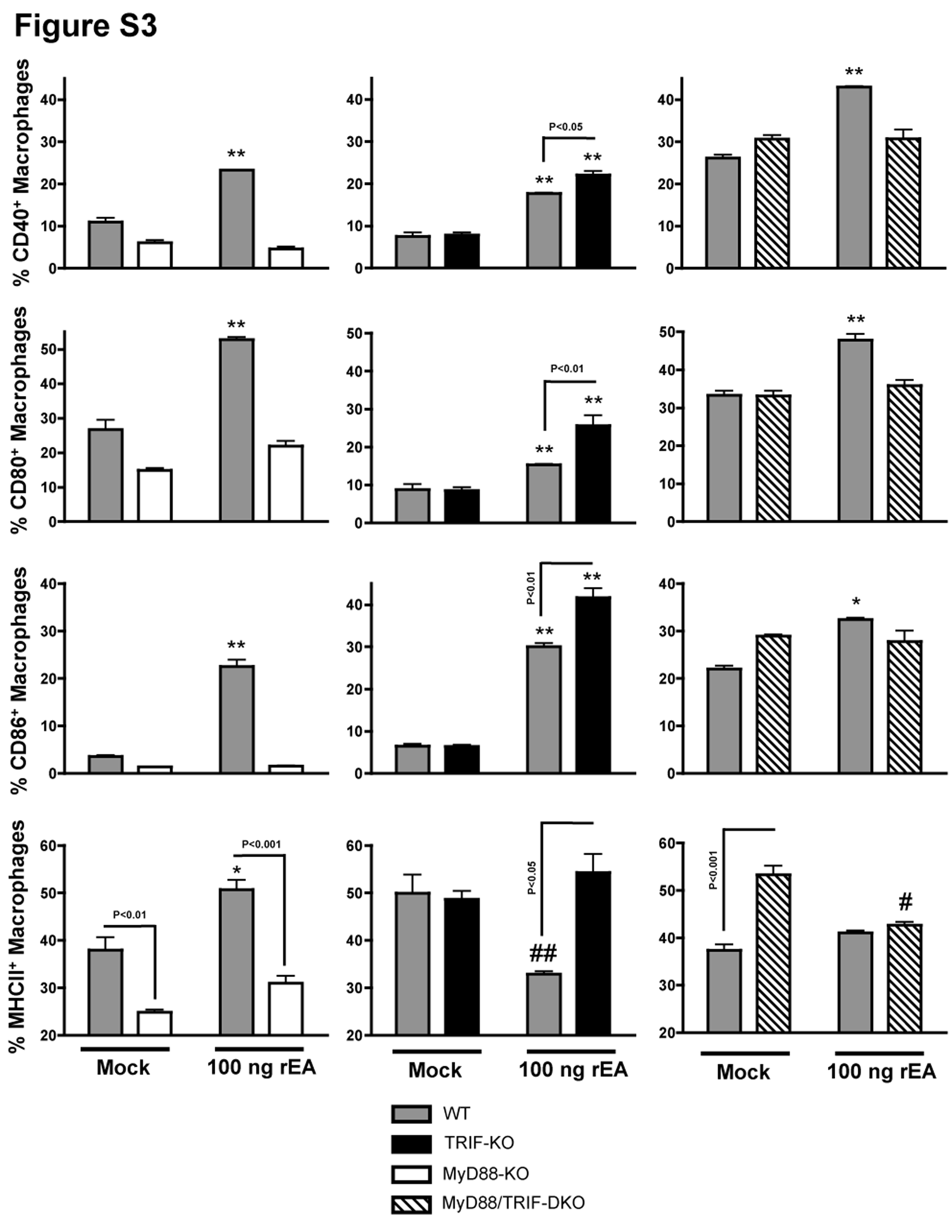

Supplement: Figure S3 — TRIF acts as a negative regulator of rEA-induced MyD88-dependent activation of macrophages in vivo . C57BL/6 WT (N = 3–4), MyD88-KO (N = 3), TRIF-KO (N = 3–4), and MyD88/TRIF-DKO (N = 4) mice were injected with 100 ng of rEA. Splenocytes were harvested at 6 hpi, processed, stained for expression of surface markers, and FACS sorted as described in Materials and Methods. All genotype mock-injected mice (N = 2–3) were included in analysis. One of two representative experiments is shown. Separate sets of WT mice were utilized for comparison with each knockout genotype. Activation of CD11b+, CD19−, and CD3− macrophages is shown. The bars represent Mean ± SEM. Statistical analysis was completed using a two tailed homoscedastic Student's t-tests. *, ** - Indicate values significantly higher (#, ## - lower) from those in mock injected animals (of the same genotype), p<0.05, p<0.001 respectively. (TIF) [file pone.0022064.s003.tif]

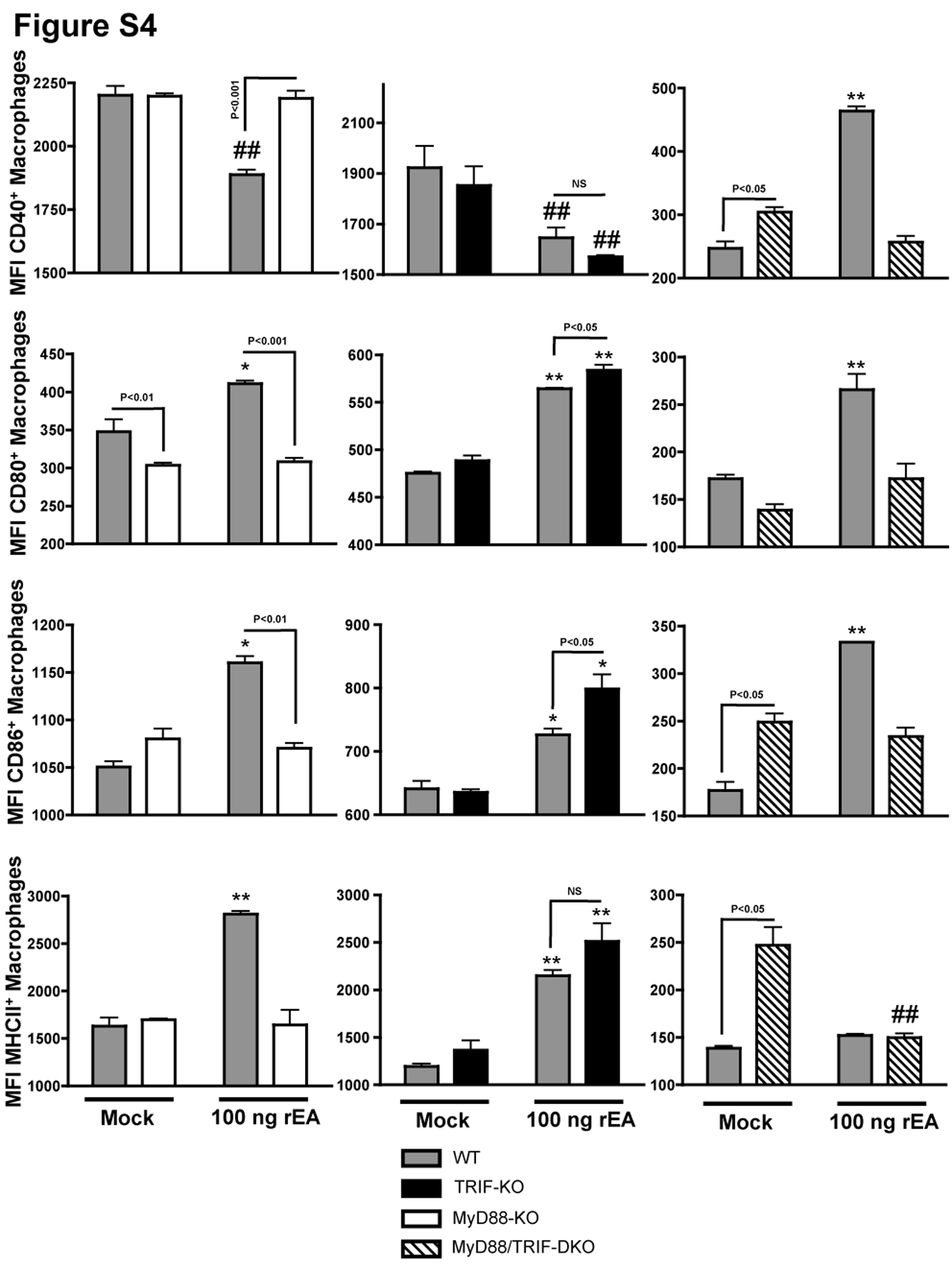

Supplement: Figure S4 — TRIF acts as a negative regulator of rEA-induced MyD88-dependent activation of macrophages cells in vivo . C57BL/6 WT (N = 3), MyD88-KO (N = 3), TRIF-KO (N = 3), and MyD88/TRIF-DKO (N = 4) mice were injected with 100 ng of rEA. Splenocytes were harvested at 6 hpi, processed, stained for expression of surface markers, and FACS sorted as described in Materials and Methods. All genotype mock-injected mice (N = 2–3) were included in analysis. Separate sets of WT mice were utilized for comparison with each knockout genotype. Mean Fluorescent Intensity (MFI) is shown and is indicative of amount of analyte per cell. The bars represent Mean ± SEM. Statistical analysis was completed using two-tailed homoscedastic Student's t-tests. *, ** - Indicate values significantly higher (#, ## - lower) from those in mock-injected animals (of the same genotype), p<0.05, p<0.001 respectively. (TIF) [file pone.0022064.s004.tif]

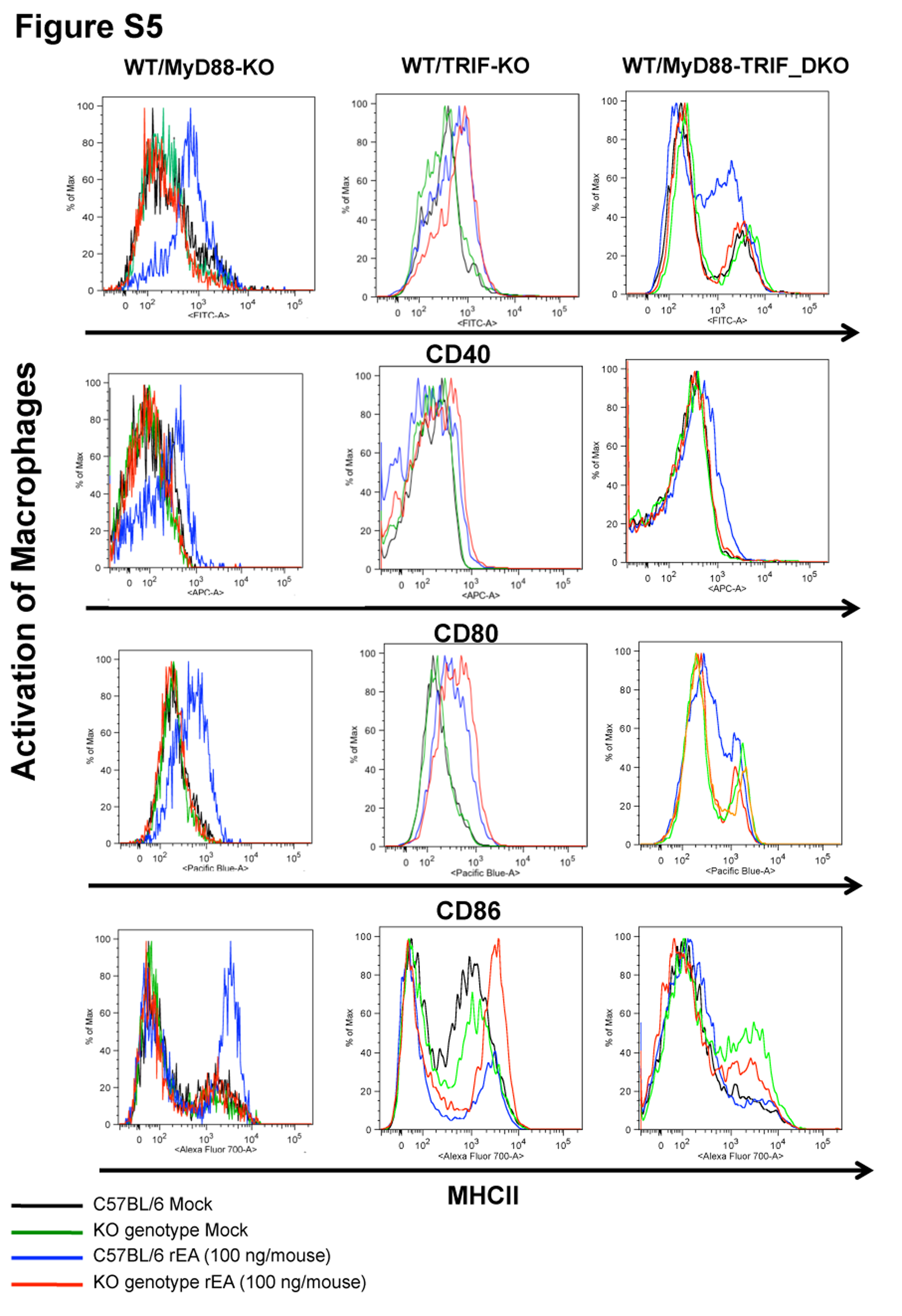

Supplement: Figure S5 — TRIF acts as a negative regulator of rEA-induced MyD88-dependent activation of macrophages in vivo . Representative histograms are illustrated on this figure. (TIF) [file pone.0022064.s005.tif]

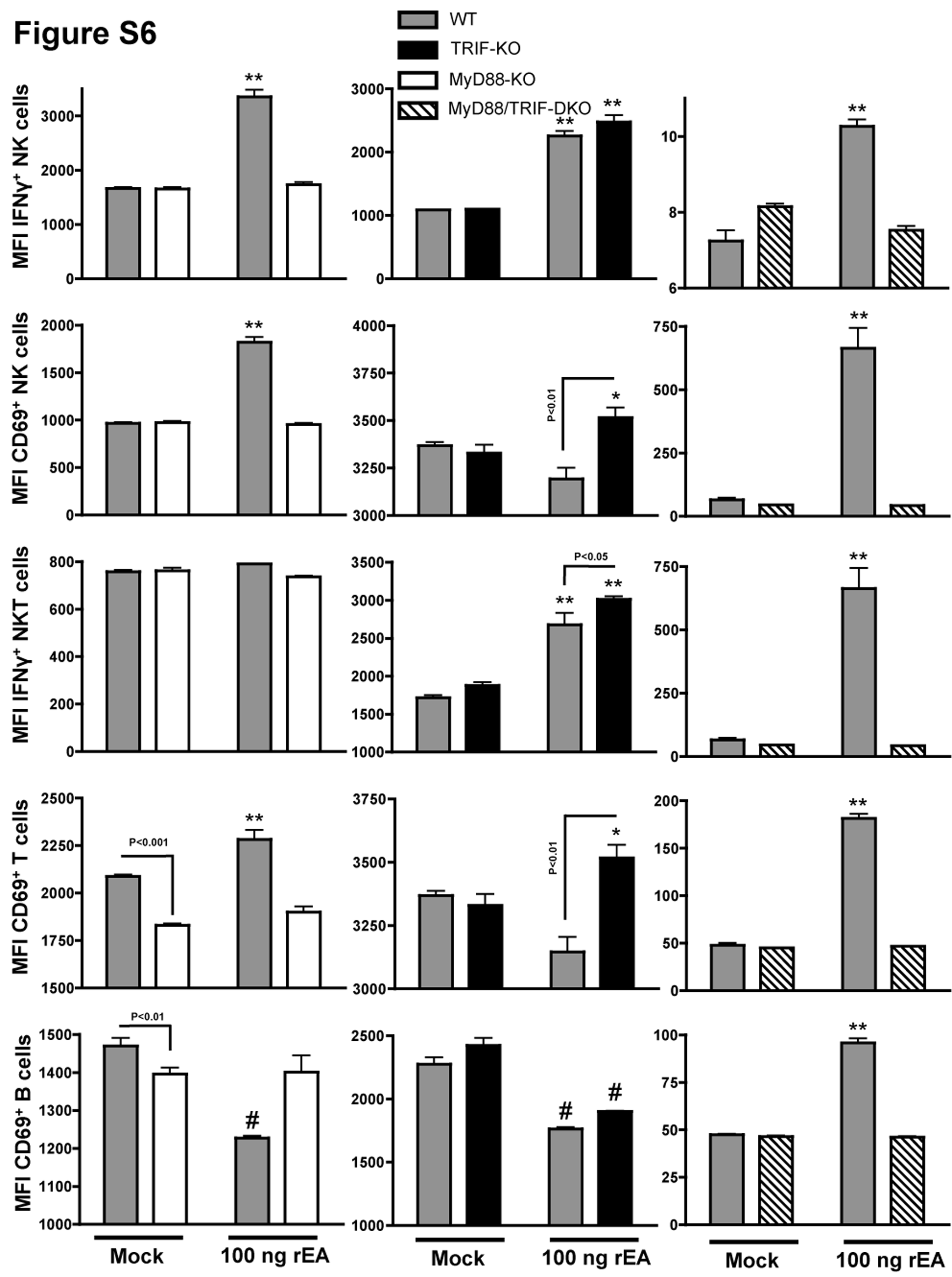

Supplement: Figure S6 — TRIF acts as a negative regulator of rEA-induced MyD88-dependent activation of NK, NKT, T, and B cells in vivo . C57BL/6 WT (N = 3), MyD88-KO (N = 3), TRIF-KO (N = 3), and MyD88/TRIF-DKO (N = 4) mice were injected with 100 ng of rEA. Splenocytes were harvested at 6 hpi, processed, stained for expression of surface markers (intracellular staining was performed for IFNγ), and FACS sorted as described in Materials and Methods. All genotype mock-injected mice (N = 2–3) were included in analysis. Separate sets of WT mice were utilized for comparison with each knockout genotype. Mean Fluorescent Intensity (MFI) is shown and is indicative of amount of analyte per cell. The bars represent Mean ± SEM. Statistical analysis was completed using two-tailed homoscedastic Student's t-tests. *, ** - Indicate values significantly higher (#, ## - lower) from those in mock-injected animals (of the same genotype), p<0.05, p<0.001 respectively. (TIF) [file pone.0022064.s006.tif]

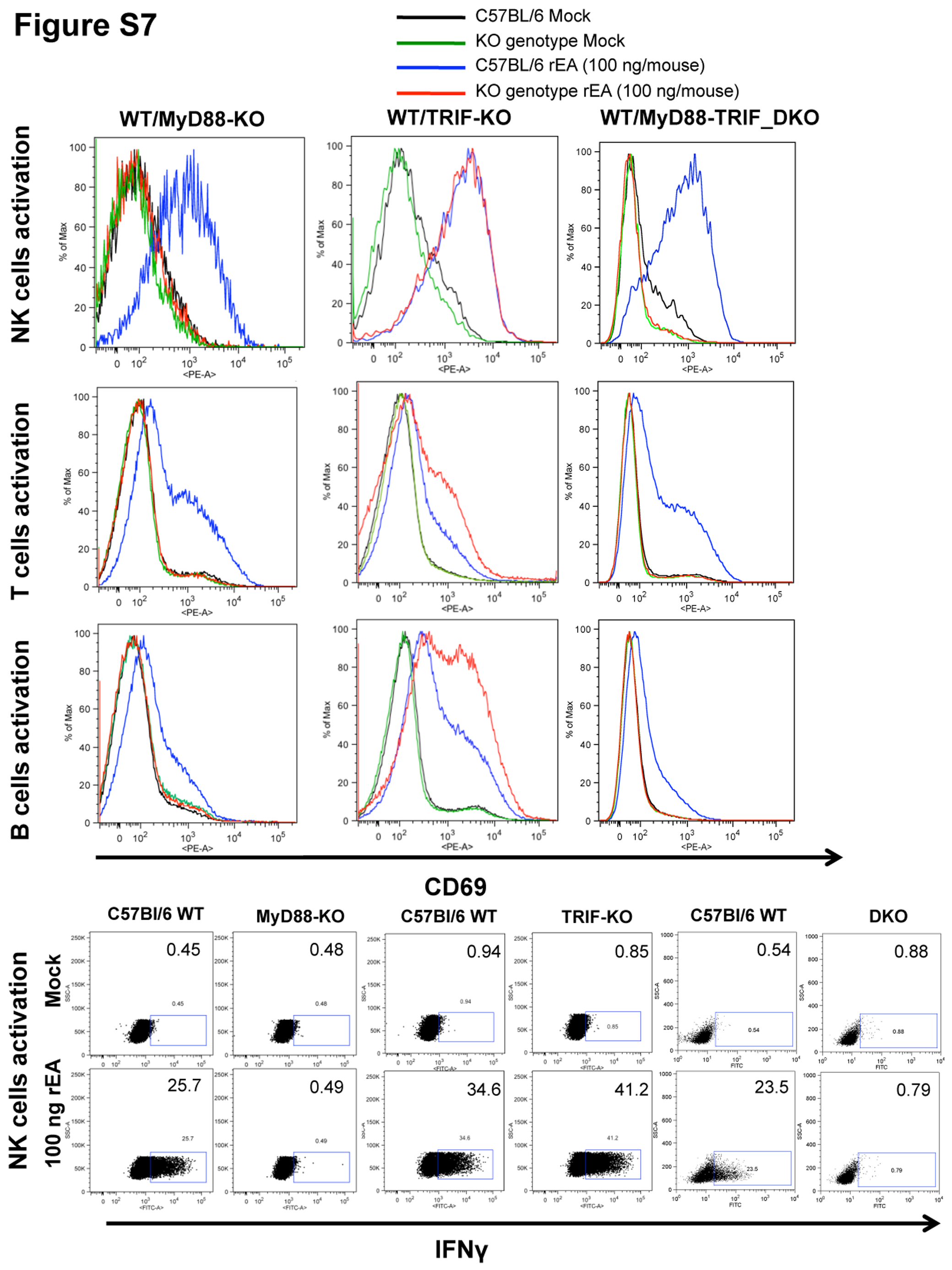

Supplement: Figure S7 — TRIF acts as a negative regulator of rEA-induced MyD88-dependent activation of NK, NKT, T, and B cells in vivo . Representative histograms and plots are illustrated on this figure. (TIF) [file pone.0022064.s007.tif]

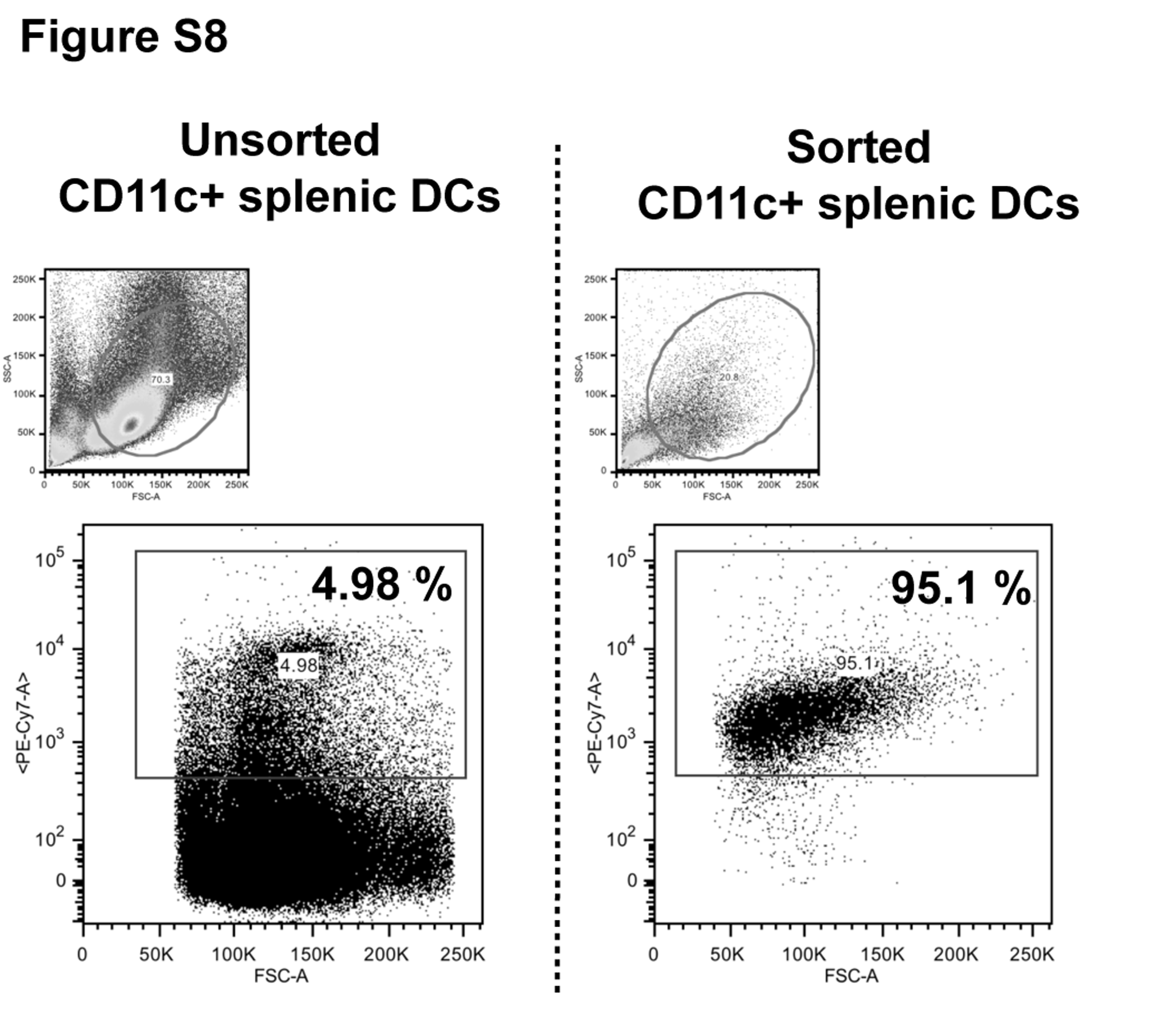

Supplement: Figure S8 — Validation of CD11c+ DCs isolation. DC sorting resulted in >95% pure CD11c positive cell population. The viability of recovered DCs was ∼90% as measured by trypan blue viability staining. (TIF) [file pone.0022064.s008.tif]
